# Capacity Building for a New Multicenter Network Within the ECHO IDeA States Pediatric Clinical Trials Network

**DOI:** 10.3389/fped.2021.679516

**Published:** 2021-07-14

**Authors:** Robert D. Annett, Scott Bickel, John C. Carlson, Kelly Cowan, Sara Cox, Mark J. Fisher, J. Dean Jarvis, Alberta S. Kong, Jessica S. Kosut, Kurtis R. Kulbeth, Abbot Laptook, Pearl A. McElfish, Mary M. McNally, Lee M. Pachter, Barbara A. Pahud, Lee A. Pyles, Jennifer Shaw, Kari Simonsen, Jessica Snowden, Christine B. Turley, Andrew M. Atz

**Affiliations:** ^1^Department of Pediatrics, University of Mississippi Medical Center, Jackson, MS, United States; ^2^Department of Pediatrics, University of Louisville School of Medicine and Norton Children's Hospital, Louisville, KY, United States; ^3^Department of Pediatrics, Tulane University School of Medicine, New Orleans, LA, United States; ^4^Department of Pediatrics, University of Vermont, Burlington, VT, United States; ^5^Department of Community and Public Health Sciences, University of Montana, Missoula, MT, United States; ^6^Fran and Earl Ziegler College of Nursing, University of Oklahoma Health Sciences Center, Oklahoma City, OK, United States; ^7^Dartmouth-Hitchcock Clinic: Dartmouth-Hitchcock Medical Center, Lebanon, NH, United States; ^8^Department of Pediatrics, University of New Mexico Health Sciences Center, Albuquerque, NM, United States; ^9^Department of Pediatrics, Division of Hospitalist Medicine, John A. Burns School of Medicine, University of Hawai'i at Manoa, Honolulu, HI, United States; ^10^ECHO IDeA States Pediatric Clinical Trials Network Data Coordinating and Operations Center, University of Arkansas for Medical Sciences, Little Rock, AR, United States; ^11^Department of Pediatrics, Warren Alpert Medical School, Brown University, Providence, RI, United States; ^12^College of Medicine, University of Arkansas for Medical Sciences, Fayetteville, AR, United States; ^13^Institute for Research on Equity and Community Health, Thomas Jefferson University, Newark, DE, United States; ^14^Children's Mercy Hospital - Kansas City Department of Infectious Diseases, Kansas University Medical Center, University of Missouri Kansas City, Kansas City, MO, United States; ^15^Department of Pediatrics, West Virginia University, Morgantown, WV, United States; ^16^Division of Organizational Development and Innovation, Southcentral Foundation, Anchorage, AK, United States; ^17^Department of Pediatrics, University of Nebraska Medical Center, Omaha, NE, United States; ^18^Department of Pediatric Infectious Disease, ECHO IDeA States Pediatric Clinical Trials Network Data Coordinating and Operations Center, University of Arkansas for Medical Sciences, Little Rock, AR, United States; ^19^Department of Pediatrics, Medical University of South Carolina, Charleston, SC, United States

**Keywords:** clinical trials, ISPCTN, pediatrics, network, research capacity building

## Abstract

**Introduction:** Research capacity building is a critical component of professional development for pediatrician scientists, yet this process has been elusive in the literature. The ECHO IDeA States Pediatric Clinical Trials Network (ISPCTN) seeks to implement pediatric trials across medically underserved and rural populations. A key component of achieving this objective is building pediatric research capacity, including enhancement of infrastructure and faculty development. This article presents findings from a site assessment inventory completed during the initial year of the ISPCTN.

**Methods:** An assessment inventory was developed for surveying ISPCTN sites. The inventory captured site-level activities designed to increase clinical trial research capacity for pediatrician scientists and team members. The inventory findings were utilized by the ISPCTN Data Coordinating and Operations Center to construct training modules covering 3 broad domains: Faculty/coordinator development; Infrastructure; Trials/Research concept development.

**Results:** Key lessons learned reveal substantial participation in the training modules, the importance of an inventory to guide the development of trainings, and recognizing local barriers to clinical trials research.

**Conclusions:** Research networks that seek to implement successfully completed trials need to build capacity across and within the sites engaged. Our findings indicate that building research capacity is a multi-faceted endeavor, but likely necessary for sustainability of a unique network addressing high impact pediatric health problems. The ISPCTN emphasis on building and enhancing site capacity, including pediatrician scientists and team members, is critical to successful trial implementation/completion and the production of findings that enhance the lives of children and families.

## Introduction

Clinical trial funding has historically been confined to large academic centers with largely urban populations and limited age groups of children (1). Likewise, populations under-represented in pediatric trials often are rural, medically underserved, and economically disadvantaged (2). Involvement of medically underserved and rural populations is critical to addressing health conditions affecting the most vulnerable populations of children in the country. These groups often have high rates of infant mortality (3), asthma (4), and childhood obesity (5).

**Figure 1:**
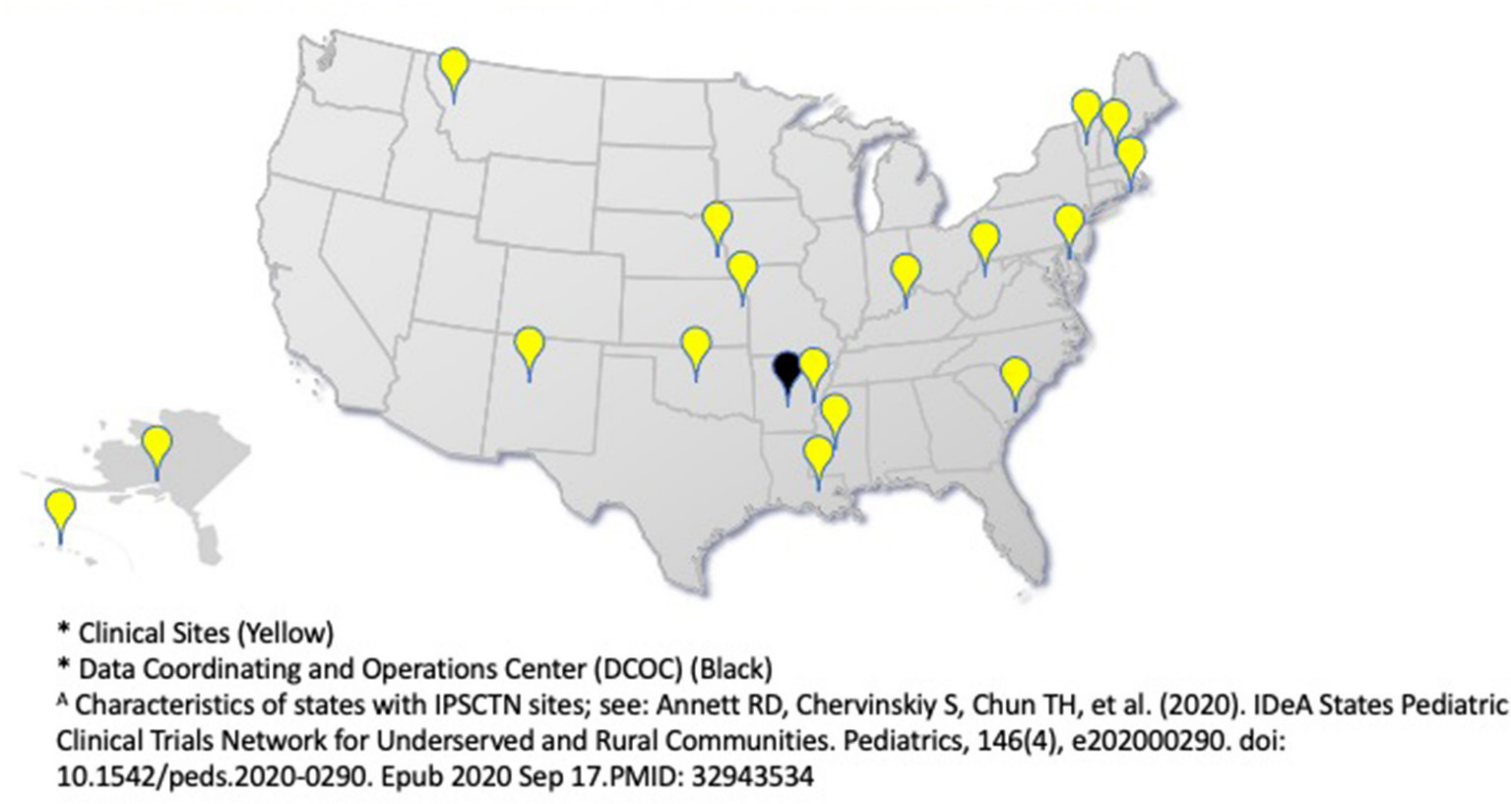
ECHO IDeA States Pediatric Clinical Trails Network 17 Clinical Sites and DCOC^*A^.

The ECHO IDeA States Pediatric Clinical Trials Network (ISPCTN), funded and established by the National Institutes of Health (NIH) in 2016 as a component of the NIH Environmental Influences on Child Health Outcomes (ECHO) program, is unique in its geographic composition and diverse in its ethnic and racial makeup (Figure 1). Characterization of these differences have recently been published (6). Clinical trial networks, such as the ISPCTN, represent an effective, efficient, and cost effective method for the creation of high quality, generalizable research (7). Networks typically consist of formal arrangements between individuals, institutions, and key stakeholders designed to facilitate the development, implementation, operation and completion of clinical trials (8, 9). As a new network charged to produce impactful pediatric research, building research capacity among sites was an initial ISPCTN priority to ensure that the nascent network could meet the challenges of conducting state-of-the-art research for underserved pediatric populations.

Capacity building has been defined as “a process of individual and institutional development which leads to higher levels of skills and greater ability to perform useful research” (10). Within the ISPCTN, building capacity was broadly operationalized to include faculty/coordinator development, enhancement and expansion of infrastructure, and enrichment of trials/research concept development (Table 1). These broad domains align with existing literature (11, 12). Additional elements crucial for long term success include building a research culture, providing mentorship, developing mechanisms for results dissemination, and supporting ongoing sustainability (8, 13–16). Extant literature largely focuses upon capacity building for allied health professionals or capacity building in global health settings (14, 17–20). Unfortunately, limited information exists regarding building research capacity for pediatric clinical trial operations (9, 21–24).

**Table 1:** Initial site capacity inventory domains.

**Domain**	**Inventory content description**
Faculty/coordinator development	• Research experience within the ECHO priority areas ° Upper and lower airway disease ◦ Pediatric obesity ◦ Neurodevelopment ◦ Positive child health ◦ Pre-, peri-, and post-natal outcomes • Recruitment experience in special pediatric disease populations and communities
Infrastructure	• Human subjects review • Study monitoring • Available laboratories • Facilities and equipment • Electronic/mobile health communication • Data management
Trials/research concept development	• Domains for trainings developed and implemented by the DCOC • Site-specific research capacity building activities
**Additional capacity inventory domains initiated by sites**	**Description**
Mentorship	• DCOC provided content • Site-specific research capacity building activities
Research Culture	• Site-specific research capacity building activities
Dissemination of results	• DCOC provided content • Site-specific research capacity building activities
Sustainability	• DCOC provided content

What can be determined from the existing literature, however, is that several barriers to building research capacity include a lack of funding, insufficient physical resources, limited research experience and expertise, competing priorities, administrative barriers, and lack of time for faculty and coordinators (8). To overcome barriers and achieve the goal of building pediatric trials capacity, an “all teach-all learn” model (25) integrated capacity building activities and locally developed training modules across all sites within this single award. The all teach-all learn model arose from quality improvement work and supports bidirectional learning, particularly focused on community health improvement (26).

Here we aim to describe findings from an ISPCTN pediatric research capacity inventory and to highlight the parallel development of a professional development curriculum, as well as qualitative reports of site-specific learning activities aimed at enhancing pediatric research capacity. Three primary capacity domains are presented: faculty/coordinator development, enhancement and expansion of organization-institutional infrastructure, and clinical trials/research concept development.

## Materials and Methods

### Assessment Inventory

In the 1st year of ISPCTN, each awardee site principal investigator and affiliated sites were sent a REDCap site assessment inventory, developed by the Data Coordinating and Operations Center, in December 2016 (~2 months after sites received initial funding). The inventory was completed by each site and affiliated site(s) prior to Network trial initiation. The domains of the inventory sought to identify and describe existing infrastructure and was used as a tool to inform the overall capacity building needs of the Network prior to trial initiation, thus did not meet the 45 CRF 46 definition of research. The inventory consisted of 57 multiple-choice and open-ended questions.

Inventory domains and description of content questions are outlined in Table 1. Special pediatric populations were ascertained using the total reported number of active pediatric patients seen at each site annually. Sites then reported subgroups from ECHO priority areas (airway, obesity, neurodevelopment, prenatal/perinatal/postnatal, positive child health) and patient demographic characteristics. Recruitment capacities at sites were characterized by languages spoken at associated clinics, need and ability to provide multi-language recruitment materials, hours of operation, recruitment methods and requirements needed for recruitment activities.

Other site capacities were inventoried. These included a human subjects review domain that ascertained information regarding regularity of Institutional Review Board (IRB) meetings and possible obstacles to timely reviews. The study monitoring domain collected site information including location of source documents stored in medical records and the ability to accommodate monitor visits, including work space and access to medical record (paper/electronic). The laboratory domain assessed site access to a local laboratory for specimen processing and dedicated equipment (e.g., centrifuge, refrigerator, and freezer). Sites were also assessed for imaging capabilities, including the availability of pediatric facilities for X-ray and MRI.

Facilities questions elicited information on infrastructure available for research, including neonatal intensive care unit (NICU) presence at the site, dedicated pediatric research space, investigational pharmacy, storage for lab supplies, practice management system, and medical records. Information was obtained on electronic/mobile health communication and if sites were tracking mobile device usage in their patient community. The data management domain included the availability of EHR resources.

### Curriculum Development

Professional development curriculum and site-developed training modules were created by the DCOC. The professional development curriculum was comprised of learning themes (Table 2). These were developed from DCOC expert input, based upon research trainings offered through the Arkansas Translational Research Institute and guided by the ISPCTN mission that includes engaging rural and underserved communities. Thus, core learning themes included: clinical trials essential elements, Institutional Review Board/ethics/regulatory teachings, data management, and community engagement. To further foster the development of pediatric scientists, a learning theme providing opportunities for interaction with a pediatric researcher was implemented. Finally, several specific professional development offerings were created from participant requests.

**Table 2:** DCOC led faculty and coordinator development trainings.

**Domain and topic**	**Training focus**^**a**^	**Participant total**	**Capacity building domain**^**b**^
**30 min with a research mentor series**			
• 30 min with a research mentor–pediatric faculty 1	I/S	4	M
• 30 min with a research mentor–pediatric faculty 2	I/S	16	M
• 30 min with a research mentor–pediatric faculty 3	I/S	13	M
• 30 min with an ISPCTN researcher–pediatric faculty 4	I/S	18	M
• 30 min with a research mentor: PK research–pediatric faculty 5	I/S	28	M
• 30 min with a research mentor–pediatric faculty 6	I/S	14	M
**IRB/ethics/regulatory**			
• Single IRB & role of the IRB in pediatric research	H	37	I
• Pediatric assent and ethical considerations	H	45	I
• Understanding the effects of health literacy on informed consent	H	26	I
• The ethics of human subjects research	H	32	I
• Informed consent (module only)	H	3	I
• The influences of health literacy on clinical research trials	H	22	TCD
**Community engagement**			
• Community engagement in clinical research	R	31	TCD
• Disparities affecting children among the American Indian communities	R	25	TCD
• Conducting clinical trials in rural populations	R	47	TCD
• Role a community advisory board plays in your research study	I/S	51	TCD
**Clinical trials essentials**			
• Overview of data safety monitoring boards	S	28	F/CD
• Research record keeping: essential practices for your research team	S	69	I
• Phases of a clinical trial	S	10	F/CD
• Protocol deviations (module only)	S	1	I
• Research misconduct	S	7	F/CD
• Emergency use of investigational products and devices	S	24	TCD
**Data management**			
• Data definition and data management	D	22	I
• Data management at clinical sites	D	19	I
• Reliable data collection	D	16	I
**Other**			
• Advertising your research study	E	24	I
• Lessons learned from the protocol review committee	I/S	27	I
• Expanded access to investigational drugs	I/S	40	TCD
• Writing for publication	I/S	55	D
• Science departments & science advocacy	I/S	1	RC
• Write winning grant proposals	I/S	8	S

a *Primary Training Focus: I/S, Investigator/Staff; R, Recruitment; H, Human subjects; S, Study monitoring; L, Laboratories, facilities and equipment; E, Electronic/Mobile health communication; D, Data management*.

b *Capacity Building Domains: F/CD, Faculty/Coordinator Development; I, Infrastructure; TCD, Trial/Research Concept Development; M, Mentorship; RC, Research Culture; D, Dissemination of results; S, Sustainability*.

## Results

### Inventory Findings

Overall, 17 ISPCTN sites and 7 affiliates are predominantly at academic medical centers (83%; 20 of 24 total responses), with sites also including Tribal health organizations and primary care centers.

### Faculty and Coordinator Development

All ISPCTN site principal investigators reported having clinical trials expertise [100% (*n* = 24) reporting previous experience with clinical trials] in ECHO disease priority domains; with 17 of 24 principal investigators reporting participation in a clinical trials network. However, variability was observed. ECHO domains with the greatest site investigator trial expertise were perinatal outcomes (*n* = 17), obesity (*n* = 17), and airway diseases (*n* = 18) (range 71–75% of investigators reporting trial experience in these domains). Less trial experience was observed in positive child health and neurodevelopment [67% (*n* = 16) and 50% (*n* = 14) of investigators reporting trial experience, respectively]. Among study coordinators, less trial experience in ECHO domains was reported [38–42% (*n* = 9–10) with previous experience].

### Experience With Recruitment Approaches

A broad range of recruitment approaches were identified. Most popular methods were flyer, mailings and attending health fairs, with 71–88% (*n* = 17–21) of sites favoring these approaches. Recruitment methods utilized by <10% of sites included: provider recruitment (*n* = 2), referral from hospital/clinic staff (*n* = 2), websites (*n* = 2), 3rd party recruitment companies (*n* = 1), patient registries (*n* = 1), e-newsletters (*n* = 1), word of mouth (*n* = 1) and Instagram© (*n* = 1). E-media use included Facebook© (54%; *n* = 13) and Twitter© (25%; *n* = 6).

### Affiliate Needs

Knowledge gaps in regional affiliates that some sites had partnered with to promote clinical trials recruitment were identified. Local study conduct procedures and follow-up education were identified to increase the number of trained clinical investigators and research coordinators conducting clinical trials for children in rural and underserved communities.

### Infrastructure (Facilities and Equipment)

Most ISPCTN sites had facilities critical to implementation of pediatric trials. NICUs were identified at 79% (*n* = 19) of sites, while other on-site facilities were frequently present [on-site pharmacy was reported at 93% (*n* = 20) of sites; neuroimaging facilities on site ranged from 83 to 92%; *n* = 20–22]. However, research pharmacy capacity for investigational agents was reported at fewer sites (79%; *n* = 19). Infrastructure for biosample storage and shipping, research refrigerator and freezer availability, and refrigerated centrifuges were frequently reported [92–96% (*n* = 22–23) of sites].

### Access to Electronic Health Records

A majority of our sites use electronic medical records (20 of 24 total responses reporting use of electronic medical records), with EPIC and Cerner being the most common (22 of 30 sites and subsites) of those using electronic medical records.

### Electronic/Mobile Health Communication

Patient communication through email occurs at many sites (75%; *n* = 18 of 24), though text messaging is less often used (38%; *n* = 9). However, across all sites, the estimated percent of patients with an email address was 60% (median). A high level of enthusiasm was evident for using e-communication for collection of research data [96% (*n* = 23) of sites expressing interest in this modality], though relatively few actively collect information on patient mobile capabilities (25%; *n* = 6).

### Trials/Research Concept Development

The DCOC provided a curriculum in an effort to increase the capacity for investigators and coordinators to develop research concepts. Training domain, content area, training focus and number of attendees for DCOC-built modules are presented in Table 2. There is no information available on participants who viewed the archived recordings of these trainings. The range of participants for each live module varied, as these were voluntary trainings. Due to the diverse location of sites, modules included a combination of operational as well as conceptual topics. The DCOC facilitated communication and collaboration across the network sites, resulting in shared content, practices, and resources through an all teach-all learn model, which provided opportunities for bi-directional learning, as well as access national expertise for specific gaps and resource needs. These modules covered a wide range of basic and applied skills and were implemented beginning the 1st year of Network operations and into the 2nd year.

### Site-Specific Learning Activities

These learning and capacity building activities were developed utilizing a combination of ISPCTN and local site resources. Each network site focused on areas of local need, as determined by site leadership, and shared between sites and the Network. Table 3 highlights the qualitative findings of learning activities developed and conducted by sites, and grouped by the capacity building inventory domains. Network funding played a role in sites initiating interaction with local infrastructure resources to support pediatric trials. Table 3 provides more nuanced information on site generated topics including mentoring, constructing an institutional research culture and activities for promotion of sustainability.

**Table 3:** ISCPTN capacity development activities developed and conducted by sites.

**Domain**	**Site developed content**
Faculty/coordinator development	Research Boot Camp: provided a group lecture and small group tutorial sessions. Community seminars focused on concepts, practices and ethics of community outreach. General clinical trials training such as CITI, Good Clinical Practice and grants management. ISPCTN trial-specific trainings for academic pediatricians and coordinators. Pediatrics faculty-specific development plans, including research mentoring. Pediatric Trials Network opportunities to expand opportunities for early career faculty and build site capacity. Linking early career faculty to research seminars in other related departments (e.g., Maternal Fetal Medicine Research Seminar). Participation in national coordinator trainings encouraged and funded (e.g., joining Society of Clinical Research Associates: SOCRA). Clinical Research Education for the Workforce program for coordinators developed by the parent university. Participation in existing programs, including: • CITI Advanced Clinical Research Coordinator Essentials. • University of Washington Biostatistics Bootcamp. • DIA Clinical Research Fundamentals Bundle. • Northwestern University Coordinator Bootcamp
Infrastructure	Monthly meetings structured around the Joint Task Force for Clinical Trial Competency Core Competency Domains (source: https://mrctcenter.org/clinical-trial-competency/). Laboratory “scavenger” hunt to foster finding and engaging hard to identify people and data required to do pharmacokinetic work. Development and implementation of recording system to ensure all training requirements are met and covered prior to beginning of study. All coordinators and research staff are responsible for helping educate and cross-train new members of the research team (shadowing for study clinic visits, consenting process, lab processing, etc.). Coordinator surveys result in targeted competency areas and served to guide training sessions. • Survey created and used was based on: Global self-assessment of competencies, role relevance, and training needs among clinical research https://mrctcenter.org/clinical-trial-competency/wp-content/uploads/sites/5/2019/08/2016-12-Global-Self-Assessment-survey-publication.pdf. Competency areas were identified using the following: • Leveling the Harmonized Core Competency Framework for the Clinical Research Professional Version 3.0 https://mrctcenter.org/clinical-trial-competency/wp-content/uploads/sites/5/2019/01/2018-12-05-Core-Competencies-Leveling-Summary.pdf.
Mentorship	Pediatrics faculty-specific research mentoring to identify ISPCTN opportunities, skills training needs and junior faculty strengths. Group/individual mentoring, including: • Development and implementation of routine mentoring sessions • Structured agenda for mentoring meeting: a. Special topic of interest to junior faculty (e.g., managing a research team, preparing a budget) b. Sharing of one research success plus one goal for the next meeting; c. and Research progress update from one faculty member.
	Site investigators link early career faculty to trials in development and activation at the siter and/or within the ISPCTN. Linking mentored early career faculty with ISPCTN writing committees. Early career faculty mentored on development of scholarly work (e.g., abstract/manuscript development). Group-based mentoring in grants management, recruitment, and the development of scholarly work products (posters, presentations and manuscripts). Short-term focused mentoring on specific scholarly activity (e.g., abstract preparation and submission). For experienced coordinators, support for mentoring new coordinators at the site and across the network. Weekly team meetings used as opportunity for mentoring of faculty and coordinators.
Research culture	Outreach efforts to identify key stakeholders that build state-wide collaborations. ISPCTN investigator membership within site's Center for Clinical and Translational Research (CCTR)/Clinical and Translational Science Awards (CTSA) Program leadership. Collaboration with IDeA Clinical and Translational Science Center professional development core at the local site.
Dissemination of results	IDeA regional conference attendance and presentations. Outreach specific to the local and national American Academy of Pediatrics meetings.
Sustainability	Institution hosts a Summer Research Scholar Program for medical students between their 1st and 2nd years of medical school. Program funds students to gain exposure to basic or clinical research early in their medical school career. Pediatric residents linked with faculty to develop scholarly work linked to ISPCTN disease-specific areas of interest.
Research and trials concepts/scientific rigor	Community engagement teaching focused on community advisory boards and guidelines for feedback to pediatric researchers.

## Discussion

Developing research capacity in a new multisite network involves many intersecting priorities, including prioritization of activities at the site and Network levels. Clinical trials capacity building for the ISPCTN benefited from guidelines in the extant literature (13, 27). The ISPCTN site assessment inventory during the initial year of funding revealed considerable pediatric-specific research capacity. This Network, including academic and non-academic sites, was led by principal investigators with clinical trials experience. Most of the Network sites had existing capacity for pediatric imaging and biosample storage facilities, as well as clinical services that could be engaged in research (e.g., NICUs). With this enhanced understanding of within and between site capacity variability, the DCOC developed and implemented training modules to complement and enhance knowledge and skills across ISPCTN faculty and coordinators in many of the identified domains needed for research skill development. As evident in Table 2, considerable efforts from the DCOC were directed to building and presentation of modules. Attendance by site faculty/coordinators suggests a high level of utilization. The skill-focused approach to capacity building, leveraging pre-existing site expertise, has allowed the Network members to build expertise more uniformity than if each site were providing local education. Having training content available across all sites, regardless of size or local expertise, formed a foundation of shared research mission and helped create shared experiences that fostered further lines of communication between site teams. Thus, key principles for capacity building, such as site-to-site collaboration, were emphasized through the inventory and subsequent training modules (27).

From our inventory we learned that capacity for pediatric trials rests upon several factors, including the type of trial (e.g., randomized controlled, pragmatic public health directed), other necessary physical attributes (e.g., presence an investigational pharmacy), and site training needs. Imperative in the inventory findings, we find three necessary elements that are of greatest importance: (a) having site leadership with trial experience; (b) providing skills-focused training for investigators and coordinators; and (c) supporting site infrastructure, such as protected time for development and conduct of trials. Our inventory provided the Network with simple metrics about sites and identified several areas where variability was evident (e.g., 17% of sites not using an electronic medical record). An inventory approach to determining site and Network capacity for clinical trials has not been evident in the extant literature on capacity building, thus the current presentation fills a gap in providing domains and context that may be a useful startup activity for new research teams and new networks. For the ISPCTN, the inventory findings were disseminated to sites in two ways: a document was developed and disseminated, and findings shared/discussed at a steering committee meeting. This approach was intended to motivate site teams to action that would facilitate the successful conduct of trials through active support and engagement in Network trials.

In the initial year of funding sites were invited to share and build upon tools and activities established, as well as draw upon and contribute to the centralized activities provided by the DCOC. The ability for sites to augment Network knowledge as a whole, and for the Network to augment the capacity of sites, was a crucial element of the capacity building approach adopted. Best practices were identified and shared, exemplifying the bi-directional commitment to accelerating pediatric trial capacity development. The ISPCTN was thus built upon existing site capacities for pediatric trials research, which could be more rapidly advanced together, while developing research capacity and Network culture, essential for successful implementation of multi-site pediatric trials.

From the assessment inventory and curriculum development, several important lessons stand out.

### Lesson 1: Research Education Matters

Educational opportunities provided by a central coordinating center, such as online training modules, provides an efficient, effective mechanism for engaging multiple sites, establishing shared operating procedures, and providing uniform knowledge for pediatric trials. Sites within the Network brought a range of individual and institutional expertise, from those sites where individuals had limited trials research knowledge and limited staff expertise, to those that had participated in networked trial groups (e.g., the Pediatric Trials Network, Pediatric Emergency Care Applied Research Network). Institutional resources for research have been supported by the ISPCTN, serving as a stimulus to support the development of pediatrician scientists. Centralized training has facilitated the development of common trials knowledge for pediatric faculty and staff. Module utilization suggests several training topics received generally greater participation (e.g., research record keeping), indicating training gaps that were not necessarily anticipated. The modules have provided a foundation that is being further built upon with the implementation of network trials and associated trial-specific trainings. We anticipate that both individual and institutional trials expertise will continue to develop as a range of pediatric trials are opened across the Network. Pediatric academic research training is a continuous and career long endeavor that needs to constantly be updated through professional development activities, which the ISPCTN recognizes and is addressing.

### Lesson 2: Assessing Local Site Resources/Experience Before Trial Launch

Characterizing infrastructure prior to the initiation of pediatric clinical trials provides necessary information, yet alone is insufficient for operationalizing trials for children in rural and medically underserved communities. This is particularly true across a network that have diverse pediatric health issues that are the focus for trials. The inventory findings suggest the need for teams with less trials research background or for those who have not received training in clinical trials need to be provided with learning opportunities that increase research skills necessary for successful trial completion. Missing in our inventory, however, was a clearer characterization of site leaders' experiences with different types of trials (e.g., industry trials, investigator-initiated trials, adaptive and pragmatic trials). While multi-site trials experience is clearly essential and important for building capacity, it is also heterogeneous, and opportunities for scholarly productivity from some trials may be limited to more seasoned investigators.

### Lesson 3: Identify Site Facilitators and Barriers to Trial Implementation

Capacity building for pediatrician scientists and coordinators must include a determination of local resources and barriers to research. Barriers may include (a) protected research time in order to establish and develop pediatrician scientists and (b) implementation of pediatric trials within rural and medically underserved communities. While this presentation addresses network capacity and development from that perspective, the ability to establish a research culture within a site and within rural communities is an important element of sustainability. Local institutions and departments developing new programs may identify administrative challenges, such as developing grant budgets and contracts. Departmental priorities for clinical productivity and teaching, as well as balancing professional commitments, can have an impact upon scholarly productivity. Similarly, building sustained relationships with underserved communities requires identification of community champions and the development of trusting relationships between investigators and community members. These and other factors (e.g., sufficient mentoring, protected time) will be important to assess in future capacity assessment inventories, as they play an indirect, but instrumental role in the success of the network.

Network capacity rests upon the sites that can successfully operationalize pediatric trials while embracing principals that support research accomplishments (27). Findings presented here provide a high-level overview of site capabilities. In order to address the mission of increasing pediatric scientists, sites developed a variety of strategies for building research skills in experienced faculty, early career pediatric faculty, coordinators, pediatric residents/fellows and medical students. A more complete understanding of the scope of the training modules, the rationale that drove local development, and the support for these local activities would be valuable in developing an understanding of the diversity of local research cultures and thus the potential for sustainability. Discussion of these activities through Network presentations (via steering committee calls and meetings) has fostered collaborative research activities. Moreover, these collaborations have resulted in scientific presentations, such as those through IDeA regional conferences and academic pediatric national/international meetings.

## Limitations

Research capacity building for multi-site pediatric clinical trials has been inadequately described in the literature. While our presentation serves to increase available information, there are several shortcomings with our approach. First, our approach identified research infrastructure capacities across sites, yet did not specifically focus on pediatric research *needs assessments* of individual sites and individual investigator research needs. Rather than an inventory, a needs assessment encompassing early career, senior faculty, and coordinators could provide greater depth of appreciation of gaps within and across sites. Additionally, sites did not provide their full educational and training materials, but may have provided topics that were unique or demonstrated a particular area of interest, and were not meant to be comprehensive of their full curricula.

## Conclusions

Identifying features of ISPCTN site has been a remarkable adjunct to the competence areas that are needed in a multi-site network. Professional development has only recently been identified as a competency area in pediatrics (28). Pediatrician scientists face similar challenges in increasing their knowledge of research-specific skills, including the conduct of pediatric trials. A major emphasis in the original development of ISPCTN was not simply to develop a trials network, but to create and sustain pediatric researchers with a firm commitment to clinical trials addressing high frequency child health conditions among communities that are historically underserved and underrepresented in trials research.

Descriptions of the implementation of trials networks have typically focused upon developing network priorities and research agendas (29–32), yet have seldom addressed the capacity of the research teams to implement trials (33). Building a research network and teams have been reported to carry unique challenges and burdens (34, 35) and our experiences demonstrate the variety of needs that must be assessed and monitored over time in order to identify learning gaps that may develop at the site and/or team level. As our network matures, devoting time to continuing to develop capacity through the domains of research culture, dissemination of results, and sustainability, will be important areas of focus. Together with the ever-important need for enhancing scientific rigor, the next phase for professional development for pediatrician scientists should include measurement of qualitative indices along with trial implementation/completion and the associated scholarly work products. With the successes to date, we are confident that the ISPCTN can succeed and prosper.

## Data Availability Statement

The original contributions presented in the study are included in the article/supplementary material, further inquiries can be directed to the corresponding author/s.

## Author Contributions

RA, AK, CT, and AA were responsible of the conceptualization and design of the manuscript contents, as well as the interpretation of data. RA drafted the initial version of the manuscript. SB, JC, KC, SC, MF, JJ, AK, JK, KK, AL, PM, MM, LPa, BP, LPy, JSh, KS, JSn, CT, and AA revised and edited the manuscript. All authors approved the final version of the manuscript.

## Conflict of Interest

The authors declare that the research was conducted in the absence of any commercial or financial relationships that could be construed as a potential conflict of interest.

## Abbreviations

NIH: National Institute of Health
ECHO: Environmental Influences on Child Health Outcomes
ISPCTN: ECHO IDeA States Pediatric Clinical Trials Network
DCOC: Data Coordinating and Operations Center
IRB: Internal Review Board
MRI: Magnetic Resonance Imaging
NICU: Neonatal Intensive Care Unit
HER: Electronic Health Record.
